# The Choice of a Diploma

**Published:** 1898-09-03

**Authors:** 


### The Choice of a Diploma.

When it has been decided that medicine is to be one's career, it is a matter of no small importance, and to tell the truth it is a matter of no small difficulty, to find out the best way of entering the profession. Much ignorance on the subject prevails; the regulations are being constantly altered, schoolmasters and tutors are very apt not to see the bearing of some of the changes which are from time to time introduced; and even medical men are by no means always reliable guides, their own personal experiences often having no relation whatever to the rules and regulations now in force.

It is, then, extremely desirable that the would-be student should from the beginning have a clear conception of what it is at which he aims and of the routes which are open to him by which he may attain the goal of his desires, for it is unfortunately the fact that a very little slip in the early days of his student life may make a great difference to the sort of practice which will be open to him when he has obtained his qualications.

### The Qualification Muddle.

At the present time about twenty different examining bodies are engaged in granting medical diplomas and degrees. In nearly every case they each give qualifications of several kinds; in many cases they combine together in groups to form what are called Conjoint Examining Boards. Sometimes the resulting qualifications give a man the right to place the letters "Dr." before his name. Sometimes they do not; and, while some of the diplomas qualify for the highest hospital appointments, many are of no help at all in that direction, so that a man, after going with due credit through his curriculum and spending much time and labour in obtaining a qualification, may find himself limited to general practice, or at any rate debarred from admission to the staff of any of our great metropolitan hospitals.

### The First Thing to Decide.

The first thing, then, that the would-be student has to decide is what he wants to be. Does he wish to be a surgeon or a physician to a London hospital, or is he content to pass his life as a general practitioner? Does he want to obtain the degree of M.D., or to remain "Mr. So-and-So, Surgeon"; or will he be content to be a Licentiate of the Society of Apothecaries? All these qualifications are equal before the law; they all qualify for the army and for the poor law, but they do not qualify equally for hospital appointments, and thus they do not equally give the *entrée* to consulting practice.

This condition of affairs is extremely unfortunate, because no young student fresh from school can tell in the slightest degree what is in him, and from sheer diffidence he may allow himself to get into a rut which leads to nowhere, and out of which it is by no means easy to escape at a later period in his career.

### The General Medical Council and the Examining Bodies.

Let us then say a word as to the meaning and status of these various diplomas. The General Medical Council is a body established by Act of Parliament for the regulation of medical education and for the maintenance of a register of legally qualified medical practitioners. It does not itself examine anyone, its duty being merely to look after the examining bodies, and to keep up the standard of the curriculum. No one is legally qualified unless he has been placed upon the register, and as the General Medical Council has decided that no one shall be placed upon the register who has not passed a satisfactory examination in all three branches of the profession—medicine, surgery, and midwifery—the Colleges of Surgeons and of Physicians have had to combine to give a conjoint diploma in their several subjects, which shall be accepted by the Medical Council. Hence the "Conjoint Examining Boards" of which we have spoken. The Universities, however, examine, in all subjects, as also do the Societies of Apothecaries in London and in Dublin. The qualifications then which admit to the register are the degrees of Bachelor of Medicine and Bachelor of Surgery in the various Universities; the conjoint diplomas of the Colleges of Physicians and of Surgeons in London, in Edinburgh and Glasgow combined, and in Dublin; and the licences of the Societies of Apothecaries.

### The Higher Qualifications.

These qualifications merely admit to the register, but the Royal Colleges of Physicians have also Members and Fellows, the Royal Colleges of Surgeons have Fellows, while the Universities grant the degrees of Doctor of Medicine and Master of Surgery, all of which are higher qualifications. It must be remembered, however, that although the lower qualifications are stepping stones to the higher in each body, the different Colleges and Universities take but little cognizance of each other's examinations, so far as their higher qualifications are concerned, so that if one wishes to avoid waste of time it is necessary to decide at once what qualification to aim at.

One might have thought that the University degrees would have stood in greatest honour, but the fact that the London hospitals generally refuse to elect as physicians any who are not members of the Royal College of Physicians of London, or as surgeons any who are not fellows of the Royal College of Surgeons of England, and the further fact that London consulting practice depends almost absolutely upon the possession of a hospital appointment, together render the possession of one or other of these two diplomas almost essential to those who wish to tread the higher walks of the profession. Hence the great desirability of arranging one's course of study, and of examinations, so as to obtain the so-called double qualification granted by the English Conjoint Board, namely the M.R.C.S. England and the L.R.C.P. London. Not that these qualifications themselves give the *entrée* to hospital appointments, but that they are necessary preliminarie to the higher diplomas, so that in taking them no time is wasted and no labour is thrown away. Moreover these diplomas have the great advantage that they do not tie a man as to his future course. Whether a man wishes to be a physician or a surgeon or a gynæcologist he is on the proper track until his "Conjoint" is passed.

If, however, he wishes to be a physician to a hospital it is a great advantage—almost indeed, although not quite, a necessity—that he should possess in addition a university degree.

### Qualifications for General Practitioners.

To a very large number of men it is clear from the beginning that the higher walks of the profession are not for them. Sometimes from lack of ambition; sometimes from that misplaced ambition which looks only to the setting up of a home at the earliest possible moment; sometimes from obvious intellectual inferiority —or shall we say peculiarity?—which makes the passing of examinations a matter of great difficulty; sometimes, again, from love of country life, men put on one side all thought of consulting work and aim exclusively at general practice. What is the best diploma for them?

To this question we have no hesitation in answering that a university degree is by far the best qualification to obtain. However little the letters M.D. may connote to the medical eye they mean much to the public, and although it is true that in the end a man's success depends upon his merits, every little helps, and there is no doubt that most people believe that in regard to medical position the "Dr." is a better man than the "Mr." The more one is brought into contact with the ignorant, and that is unfortunately always the fate of the general practitioner, the more important do these little marks and insignia of position become.

What then about the Apothecaries' Hall? Who is to go there for a licence? Much depends upon what the L.S.A. is to call himself. If he may legally call himself "Physician and Surgeon" there seems no reason why the licence of the Society of Apothecaries should not come largely into use again as a qualification for such general practitioners as do not take a degree. In the meantime, although it is a perfectly good and respectable qualification, it has come to be looked upon as a sort of refuge for those who, from one cause or another, have missed their way in their curriculum. Not that the examinations are particularly easy, but they are sometimes more convenient for those who are driven for time, as they take place at shorter intervals than is the case at the colleges.

As far as we can see, if the L.S.A. can find for himself a satisfactory colloquial title his qualification will be a perfectly good one for general practice; but there is always this against it, which prevents our recommending anyone to aim at it alone, namely, that it leads to nothing else, so that if circumstances should alter, and an opportunity for the fulfilment of high ambitions should occur, the L.S.A. would have to go back to the beginning in his search for a higher qualification.

We have no doubt that the best all-round qualifications to take are the London conjoint, together with any M.D. that seems the most easily obtained—the London one for preference, if London work is aimed at. These qualifications are of the best for general practice, and if an opening should occur there is nothing to hold back a man who has them from the highest positions which the profession has to offer.

